# Serine Protease HTRA1 Antagonizes Transforming Growth Factor-β Signaling by Cleaving Its Receptors and Loss of HTRA1 *In Vivo* Enhances Bone Formation

**DOI:** 10.1371/journal.pone.0074094

**Published:** 2013-09-11

**Authors:** Julie R. Graham, Angela Chamberland, Qingcong Lin, X. Jian Li, David Dai, Weilan Zeng, Mark S. Ryan, Moisés A. Rivera-Bermúdez, Carl R. Flannery, Zhiyong Yang

**Affiliations:** 1 Inflammation and Remodeling Research Unit, Pfizer BioTherapeutics Research, Cambridge, Massachusetts, United States of America; 2 Global BioTherapeutic Technologies, Pfizer BioTherapeutics Research, Cambridge, Massachusetts, United States of America; 3 Immunoscience Research Unit, Pfizer BioTherapeutics Research, Cambridge, Massachusetts, United States of America; INSERM U1059/LBTO, Université Jean Monnet, France

## Abstract

HTRA1 is a member of the High Temperature Requirement (HTRA1) family of serine proteases, which play a role in several biological and pathological processes. In part, HTRA1 regulation occurs by inhibiting the TGF-β signaling pathway, however the mechanism of inhibition has not been fully defined. Previous studies have shown that HTRA1 is expressed in a variety of tissues, including sites of skeletal development. HTRA1 has also been implicated in the process of bone formation, although the precise manner of regulation is still unknown. This study investigated how HTRA1 regulates TGF-β signaling and examined the *in vivo* effects of the loss of HTRA1. We demonstrated that recombinant HTRA1 was capable of cleaving both type II and type III TGF-β receptors (TβRII and TβRIII) *in vitro* in a dose-dependent manner, but it did not affect the integrity of TβRI or TGF-β. Overexpression of HTRA1 led to decreased levels of both TβRII and III on the cell surface but had no effect on TβRI. Silencing HTRA1 expression significantly increased TGF-β binding to the cell surface and TGF-β responsiveness within the cell. To examine the role of HTRA1 *in vivo*, we generated mice with a targeted gene deletion of *HTRA1*. Embryonic fibroblasts isolated from these mice displayed an increase in TGF-β-induced expression of several genes known to promote bone formation. Importantly, the loss of HTRA1 in the knockout mice resulted in a marked increase in trabecular bone mass. This study has identified a novel regulatory mechanism by which HTRA1 antagonizes TGF-β signaling, and has shown that HTRA1 plays a key role in the regulation of bone formation.

## Introduction

HTRA1 (also called PRSS11) is a member of the High Temperature Requirement A (HTRA) family of serine proteases, which are characterized by a trypsin-like serine protease domain and at least one C-terminal PDZ domain. Mammalian HTRA1 plays a role in a variety of normal physiological processes, including protein degradation and cell signaling, and has been implicated in skeletal development and osteogenesis [1]–[3]. In addition, HTRA1 has been shown to be involved in several pathologies, in particular conditions involving aberrant deposition of extracellular matrix. For example, a single nucleotide polymorphism within the promoter region of the *HTRA1* gene has been linked to cases of age-related macular degeneration. This has been correlated to increased expression of HTRA1 protein [4], [5], which was shown to alter the choroid layer within the eye in part by cleaving fibulin 5 [6]. Mutations in the *HTRA1* gene are also believed to cause a rare disease called cerebral autosomal recessive arteriopathy with subcortical infarcts and leukoencephalopathy, commonly known as CARASIL, which is a hereditary ischemic cerebral small-vessel disease [7]. These mutations decrease HTRA1 protease activity and consequently lead to disinhibition of the transforming growth factor-β (TGF-β) family signaling. In addition to these functions, HTRA1 may affect numerous other cell processes via its regulation of TGF-β signaling, including bone formation. TGF-β plays a key role in bone remodeling by inducing osteoblast differentiation and proliferation [8] and by stimulating migration of bone mesenchymal stem cells [9]. Therefore, HTRA1-mediated modulation of TGF-β signaling may contribute to the regulation of bone homeostasis and disease processes, in particular those involving loss of bone structure.

The TGF-β superfamily of mammalian proteins consists of more than 30 structurally-related cytokines, including TGF-βs, bone morphogenic proteins (BMPs), activins, and nodals. These proteins signal through transmembrane serine-threonine kinase receptors known as type I (TβRI) and type II (TβRII) receptors. Upon ligand binding, TβRI and TβRII associate, leading to phosphorylation of TβRI by TβRII. This signaling can be enhanced by Type III TGF-β receptor (TβRIII, also called betaglycan). TβRIII is a co-receptor for TGF-β. In its membrane-bound form this co-receptor potentiates TGF-β binding to the type II receptor, thereby enhancing signaling [10], [11]. Following activation of the receptors, TβRI propagates the signal via the intracellular phosphorylation of receptor-regulated Smad proteins (R-Smads). The phosphorylated Smad complex then translocates into the nucleus where it functions as a transcription factor to regulate gene expression downstream of TGF-β signaling.

Although HTRA1 has been shown to inhibit TGF-β signaling [1], [12], [13], the exact mechanism of regulation is not fully defined. It has been previously shown that the effect of HTRA1 on TGF-β family signaling is dependent on its proteolytic activity and requires an active protease domain, however it does not appear to cleave these factors [1]. It has been reported that HTRA1 can regulate TGF-β signaling by cleaving the pro-domain of proTGF-β within the endoplasmic reticulum [12]. This intracellular mechanism does not fully explain HTRA1 regulation of TGF-β signaling however, as the addition of recombinant HTRA1 to the cell culture medium can inhibit the effects of TGF-β [1]. We believe there may be more than one point at which HTRA1 can affect the TGF-β pathway, therefore we set out to further investigate the mechanism by which HTRA1 antagonizes TGF-β signaling. Our results indicate that HTRA1 cleaves the TGF-β receptors TβRII and TβRIII, but not TβRI, resulting in a decrease in downstream signaling. Importantly, we also revealed a critical role of HTRA1 in the regulation of bone homeostasis by using targeted gene deletion. We found that deletion of *HTRA1* caused a marked increase in bone formation in mice, possibly due to the disinhibition of TGF-β family signaling.

## Materials and Methods

### Ethics Statement

All animal studies were approved and performed within strict accordance of the guidelines established by the Pfizer Institutional Animal Care and Use Committee. Mice were euthanized using carbon dioxide inhalation, and all efforts were made to minimize animal suffering.

### Cell Culture and Treatments

HeLa cells were cultured in Dulbecco’s modified Eagle’s medium (Invitrogen Life Technologies) and A549 cells were cultured in F-12K medium (American Type Culture Collection), both supplemented with 10% fetal bovine serum (FBS) and penicillin-streptomycin (Invitrogen Life Technologies). Mouse embryonic fibroblasts were prepared from embryos between 10 and 12 days gestation as previously described [14], and cultured in Dulbecco’s modified Eagle’s medium supplemented with 10% FBS, penicillin-streptomycin, and MEM non-essential amino acids (Invitrogen Life Technologies). For treatments with TGF-β, cells were transferred to serum-free medium for 24 hours and then treated with a vehicle control containing 0.4 mM HCl and 0.1% BSA or treated with TGF-β (R&D Systems) at the concentrations and for times indicated within the text.

### siRNA Transfections

Cells were transfected with 25 nM of predesigned siRNAs against either HTRA1 (Invitrogen Life Technologies) or a nonspecific negative control (QIAGEN) using either Lipofectamine RNAiMAX (Invitrogen Life Technologies) for HeLa cells or Lipofectamine 2000 (Invitrogen Life Technologies) for A549 cells according to the suggested protocol. Knockdown efficiency was assessed by western blot.

### Luciferase Assays

HeLa cells were co-transfected with either a negative control plasmid or an inducible Smad-responsive luciferase reporter plasmid from the Cignal Smad Reporter Assay Kit (SABiosciences), along with either a control plasmid (pTMED) or 0, 25, 50, 100 or 150 ng of a plasmid encoding HTRA1 (HtrA1-pTMED). Transfections were performed using FuGENE 6 (Roche Applied Science) according to the manufacturer’s protocol. Cells were incubated at 37°C for 24 hours, after which they were treated with TGF-β for 16 hours. Cells were then lysed and assayed for firefly and *Renilla* luciferase activities using the Dual-Glo Luciferase Assay Kit (Promega).

### *In vitro* Cleavage of TGF-β Receptors by HTRA1

0.5 µM purified TβRIII, TβRII, and TβRI (R&D Systems) were incubated with 0.02, 0.1, or 0.5 µM purified HTRA1 (aa 157–480) or 0.5 µM S328A (aa 157–480) with or without a synthetic peptide agonist (CPII) [15] (AnaSpec) overnight at 37°C. HTRA1 and S328A were purified as previously described [16]. Cleavage products were visualized by western blots.

### Cleavage of TGF-β Receptors from the Cell Surface

HeLa cells were transfected with either a control plasmid (pTMED) or an HTRA1 encoding plasmid (HTRA1-pTMED) in addition to plasmids encoding TβRIII (pCMV6-XL4, Origene), TβRII (pCMV6-XL4, Origene), or TβRI (pCMV6-XL, Origene) using FuGENE 6 reagent (Roche Applied Science). After 48 hours, cell surface proteins were biotinylated using 0.5 mg/mL sulfo-NHS-biotin (Pierce) for 30 min at 4°C. Cells were lysed and biotinylated cell membrane proteins were purified using streptavidin magnetic beads (Pierce). Cell surface expression of TGF-β receptor proteins was measured by western blot analysis. HTRA1 protein levels were determined by western blot analysis of conditioned medium using an anti-His antibody (Santa Cruz).

### Western Blot Analysis

Proteins were separated by electrophoresis in 4–20% Tris-Glycine gels (Invitrogen Life Technologies), transferred to a nitrocellulose membrane, and incubated with primary antibodies against TβRIII, TβRII, TβRI, cadherin (all from Cell Signaling), His-tag (Santa Cruz), Smad2/3 (BD Transduction Laboratories), phospho-Smad2 (Millipore), HTRA1 (Abgent) or β-actin (Sigma). Blots were visualized using horseradish peroxidase-conjugated secondary antibodies (Santa Cruz) and chemiluminescence (Perkin Elmer). Whole-cell extracts were prepared by lysing cells with radioimmunoprecipitation assay (RIPA) lysis buffer supplemented with protease and phosphatase inhibitor cocktails (Roche Applied Science) and then used for western analysis.

### Flow Cytometry

Cells were harvested by treating with 0.5 mM EDTA, and re-suspended in PBS supplemented with 1% BSA at a concentration of 4×10^6^ cells/ml. Cells were then incubated with either biotinylated negative control protein or biotinylated TGF-β (R&D Systems), followed by staining with avidin-FITC (R&D Systems) and Hoechst dye (Sigma Aldrich). Final flow cytometric analyses were performed on a BD FACSCanto II and processed with BD FACSDiva software.

### Quantitative RT-PCR

RNA extracts were prepared from cells using either the RNeasy Mini kit (QIAGEN) or the TaqMan Gene Expression Cells-to-Ct kit (Applied Biosystems). The relative mRNA expression levels were determined by using gene-specific TaqMan gene expression assays (Applied Biosystems). Expression levels were normalized to β-glucuronidase (GusB) and relative expression was calculated using the ΔΔC_T_ method.

### *HTRA1* Gene Targeting and Production of *HTRA1* Knockout Mice

Knockout mice used in these studies were designed and generated at Pfizer. The conditional knockout strategy was designed to flox exon 2 and 3. Gene targeting for the *HTRA1* CKO allele was carried out on 129SvEv ES cells. HTRA1-CKO targeting vector was constructed first by subcloning a genomic DNA fragment (13680 bp) with exon 2 and exon 3 roughly in the middle. Then the 5′ loxP and 3′ Neo-loxP cassettes were inserted upstream of exon 2 (501 bp from exon 2) and downstream of exon 3 (621 bp from exon 3), respectively. We first generated chimeric mice that contained the *HTRA1* CKO allele, which were mated with protamine-cre transgenic mice to generate animals that carry both the conditional allele and the protamine-cre transgene. These animals were then mated with wild-type mice to produce heterozygous exon 2 and exon 3 germline-deleted mice used for the generation of *HTRA1* null mutation mice, caused by an open reading frame shift resulting from the deletion of exon 2 and exon 3. The wild type allele was identified by PCR using a forward primer “For” (5′-CTGCATGTCCCTGTGCTCAGTTG-3′) and a reverse primer “Rev” (5′-GAACTGGGACCTTTCCAGTCCTCTTG-3′). The knockout allele was identified using the primer “For” and a knockout reverse primer “KO Rev” (5′-CCCTGCTTCTGAGTTACAGGCTAATGG-3′).

### Microcomputed Tomography

High-resolution microcomputed tomography (µCT) was used to evaluate trabecular volume fraction and microarchitecture in the distal femur (µCT20; Scanco Medical AG, Basserdorf, Switzerland) and the fourth lumbar vertebra (µCT40; Scanco Medical AG) [17]. The femur was scanned at 35 kEv with a slice increment of 9 µm. CT images were reconstructed with an isotropic voxel size of 9 µm, and the gray-scale images were segmented using a constrained three-dimensional (3D) Gaussian filter (σ = 0.8, support = 1.0) to remove noise, and a fixed threshold (35% of maximal gray scale value) was used to extract the structure of mineralized tissue. Scanning was started approximately at the growth plate and extended proximally for 200 slices. Morphometric analysis was performed on 135 slices extending proximally beginning with the first slice in which the femoral condyles had fully merged. The entire fourth lumbar vertebra was scanned at 55 kEv, with a slice increment of 12 µm, and CT images were reconstructed with an isotropic voxel size of 12 µm. The gray-scale images were segmented using a constrained 3D Gaussian filter (σ = 0.8, support = 1.0) to remove noise, and a fixed threshold (22% of maximal gray scale value) was used to extract the structure of mineralized tissue. The trabecular bone within the vertebral body (excluding regions near the endplates) was identified using manually drawn contouring algorithms on approximately 200 CT slices per vertebrae (∼2.5 mm of vertebral height). Morphometric parameters computed for both skeletal sites included the bone volume fraction (BV/TV, %), trabecular thickness (Tb.Th, µm), trabecular number (Tb.N, #/mm) and trabecular separation (Tb.Sp, µm). Tb.Th, Tb.N, and Tb.Sp were computed using algorithms that do not rely on assumptions about the underlying trabecular structure [17]–[20].

### Statistical Analyses

All results are expressed as the mean ± s.e.m. Statistical comparisons for flow cytometry and gene expression studies were made using an unpaired one-tailed Student’s *t* test. Data obtained from the µCt were analyzed for significance using ANOVA and Fisher’s protected least significant difference test.

## Results

### HTRA1 Inhibits TGF-β Signaling

**Figure 1:**
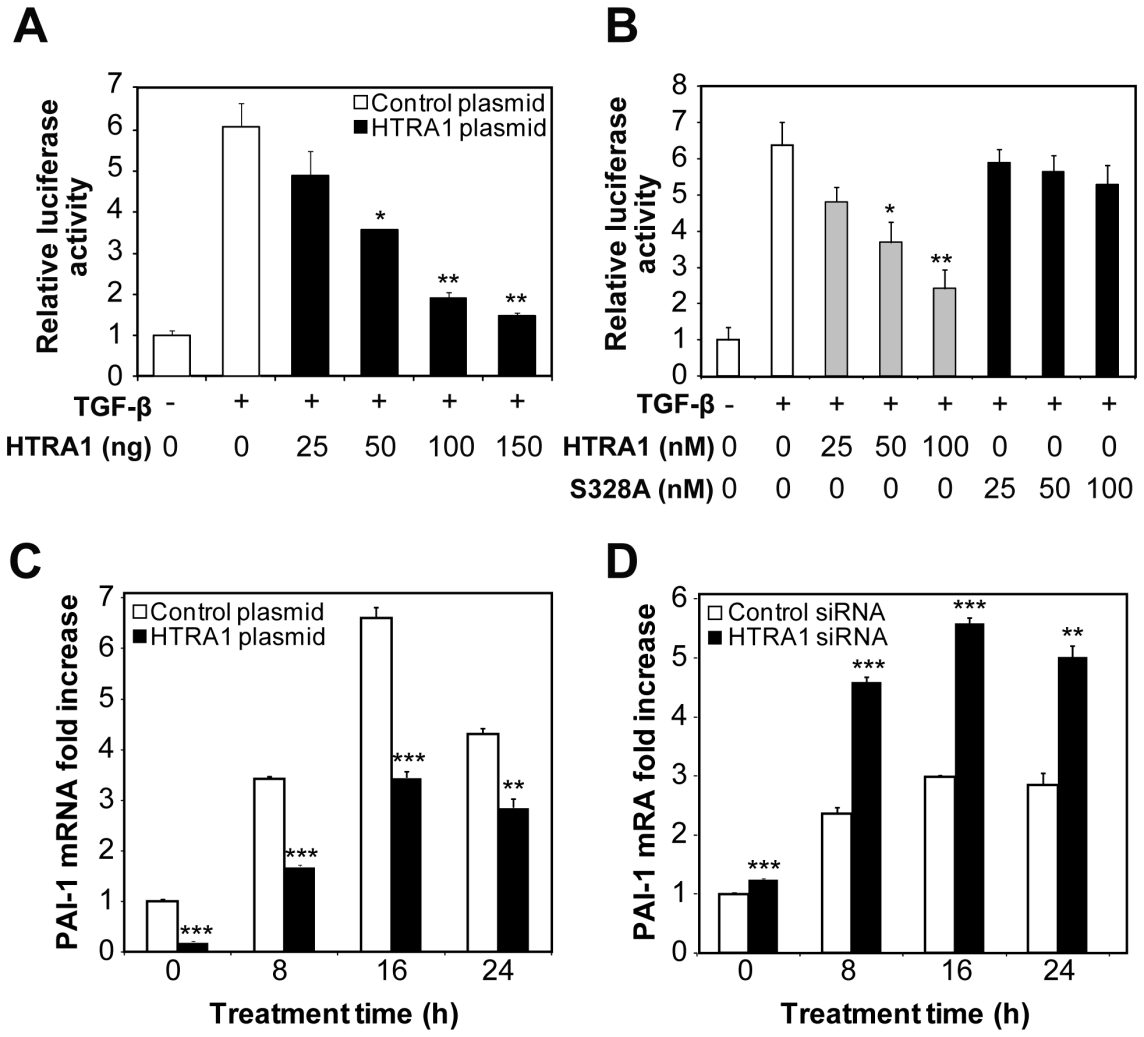
HTRA1 inhibits TGF-β-mediated transcription. (*A*) The effect of HTRA1 on TGF-β transcription was examined by co-transfecting HeLa cells with a luciferase reporter plasmid driven by tandem Smad responsive elements and with either control plasmid or 0, 25, 50, 100 or 150 ng of a plasmid that overexpresses HTRA1. After 24 hours, cells were either left untreated or treated with 10 ng/ml TGF-β for 16 hours to induce the Smad-responsive reporter gene. Relative luciferase activity was determined by calculating the ratio of firefly and *Renilla* luciferase activities. (*B*) The effect of exogenous treatment of HTRA1 on TGF-β-mediated transcription. HeLa cells were transfected with a Smad-responsive reporter and then either left untreated or treated with the indicated concentrations of purified HTRA1 or an inactive mutant, S328A. Relative luciferase activity was determined by calculating the ratio of firefly and *Renilla* luciferase activities. (*C*) TGF-β-induced expression of *PAI-1* mRNA. HeLa cells were transfected with either a control plasmid or an HTRA1 plasmid, and then treated with 10 ng/ml of TGF-β for the indicated times. (*D*) Expression of *PAI-1* mRNA in HeLa cells transfected with either a nonspecific control siRNA or with HTRA1 siRNA, then treated with 10 ng/ml of TGF-β for the indicated times. *P<0.05, **P<0.01, ***P<0.001, compared to TGF-β treated control (*A*, *B*), or empty plasmid and non-specific siRNA controls (*C*, *D*). Student’s *t*-test, n = 3.

We first wanted to establish that HTRA1 inhibits TGF-β signaling. Our initial step was to verify that HeLa cells were responsive to TGF-β and could be used as a model cell type. In accordance with previous studies [21]–[23], treatment of cells with TGF-β resulted in activation of the TGF-β pathway, indicated by an increase in Smad2 phosphorylation (Figure S1). To measure the effect of HTRA1 on TGF-β signaling, we used a TGF-β-specific transcriptional reporter assay. Cells were transfected with a construct containing the luciferase gene with Smad-responsive elements located within its promoter region, which measures activation of the TGF-β pathway. Treatment with recombinant TGF-β caused an approximate 7-fold upregulation of luciferase gene expression, which was inhibited in a dose-dependent manner by overexpressing HTRA1 (Figure 1A). Exogenous treatment of the cells with purified HTRA1 protein also inhibited TGF-β signaling in a dose-dependent manner, indicating that this regulation occurred extracellularly. A proteolytically inactive HTRA1 mutant (S328A) that contains a serine to alanine mutation within the serine protease domain [24] had no effect on TGF-β-dependent transcription (Figure 1B). These results show that HTRA1 represses TGF-β signaling, and this inhibition is dependent on the protease activity of HTRA1.

To confirm that HTRA1 was inhibiting TGF-β in a physiological context, we next examined the effect of either overexpressing or silencing HTRA1 on the expression of *PAI-1*, a well-known endogenous TGF-β-responsive gene [25]. As shown in Figure 1C, cells that overexpressed HTRA1 exhibited a decreased response to TGF-β, indicated by the decrease in *PAI-1* expression in response to TGF-β treatment. When cells were transfected with HTRA1 siRNA, which reduced HTRA1 expression as shown by western analysis (Figure S2A), the TGF-β-induced *PAI-1* levels increased as compared to cells transfected with a negative control (Figure 1D). Similar results were seen with other TGF-β -responsive genes, *Smad7* and *GADD45B* (data not shown). These data further indicate that HTRA1 negatively regulates gene expression induced by TGF-β.

### HTRA1 Cleaves Type II and III, but Not Type I, TGF- β Receptors

**Figure 2:**
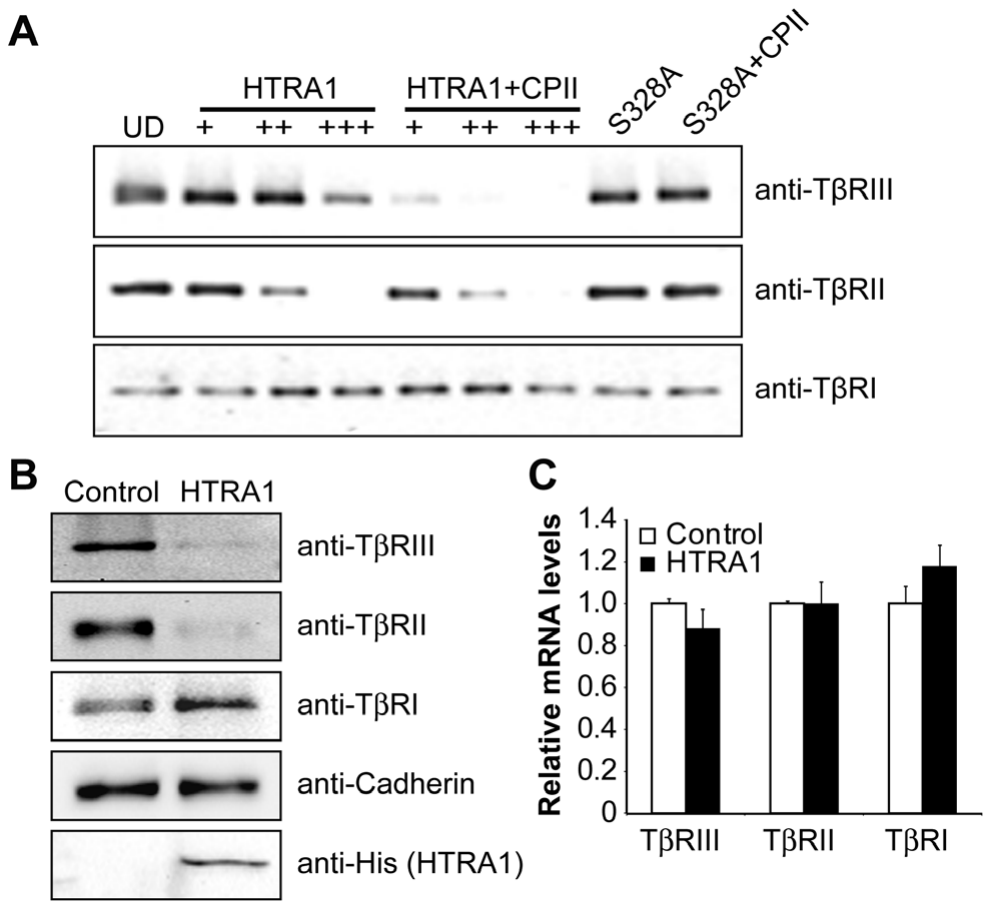
HTRA1 cleaves TGF-β receptors. (*A*) Cleavage of TGF-β receptors by HTRA1 *in vitro*. Equal amounts of purified TGF-β receptors were incubated with increasing amount of purified HTRA1 with or without CPII, an HTRA1 agonist. The inactive mutant, S328A, was also incubated with the receptors in the presence or absence of CPII. Protein digestions were analyzed by western blot. UD = undigested proteins. (*B*) HTRA1 cleavage of TGF-β receptors from the cell surface. HeLa cells were transfected with either a control or HTRA1 plasmid in addition to plasmids encoding either TβRIII, TβRII or TβRI. Membrane proteins were isolated and analyzed by western blot for cleavage of membrane bound receptors. Cadherin was used as a loading control. Overexpression of His-tagged HTRA1 was confirmed with an anti-His antibody. (*C*) mRNA levels of the TGF-β receptors after HeLa cells were transfected with either a control or HTRA1 plasmid.

We found that exogenous treatment of HTRA1 inhibited TGF-β signaling (Figure 1B), which cannot be explained by the intracellular cleavage of TGF-β by HTRA1, a mechanism previously described by Shiga et al. [12]. We therefore investigated whether another regulatory mechanism exists that involves the HTRA1-mediated degradation of an extracellular component(s) of the TGF-β signaling pathway. Incubation of mature TGF-β protein with various concentrations of recombinant HTRA1 did not cause any degradation of TGF-β (data not shown). We then determined whether HTRA1 can cleave TGF-β receptors, since the responsiveness of a cell to TGF-β can be modulated by regulating the presentation of TGF-β receptors on the cell surface. Indeed, exogenous treatment with HTRA1 resulted in cleavage of both TβRII and TβRIII in a dose-dependent manner, but it did not affect the integrity of TβRI (Figure 2A). Importantly, cleavage increased with the addition of the HTRA1 agonist CPII [15], however the proteolytically inactive S328A mutant had no effect (Figure 2A). To determine if HTRA1 can cleave membrane-bound TGF-β receptors and if cleavage can occur within the context of the cell, we overexpressed HTRA1 and prepared membrane protein extracts. Consistent with the above results, there was less TβRII and TβRIII on the surface of cells overexpressing HTRA1 as compared to the control, whereas the level of TβRI remained unchanged (Figure 2B). Overexpression of HTRA1 did not affect the mRNA expression of the TGF-β receptors (Figure 2C), therefore the decrease in protein levels on the cell surface is not due to a reduction in transcription of the receptor genes. Our data demonstrate that HTRA1 cleaves TβRII and TβRIII from the cell surface, potentially leading to a decrease in downstream TGF-β signaling.

### Silencing of *HTRA1* Expression Results in Increased Binding of TGF- β to the Cell Surface and Enhanced TGF- β Signaling

**Figure 3:**
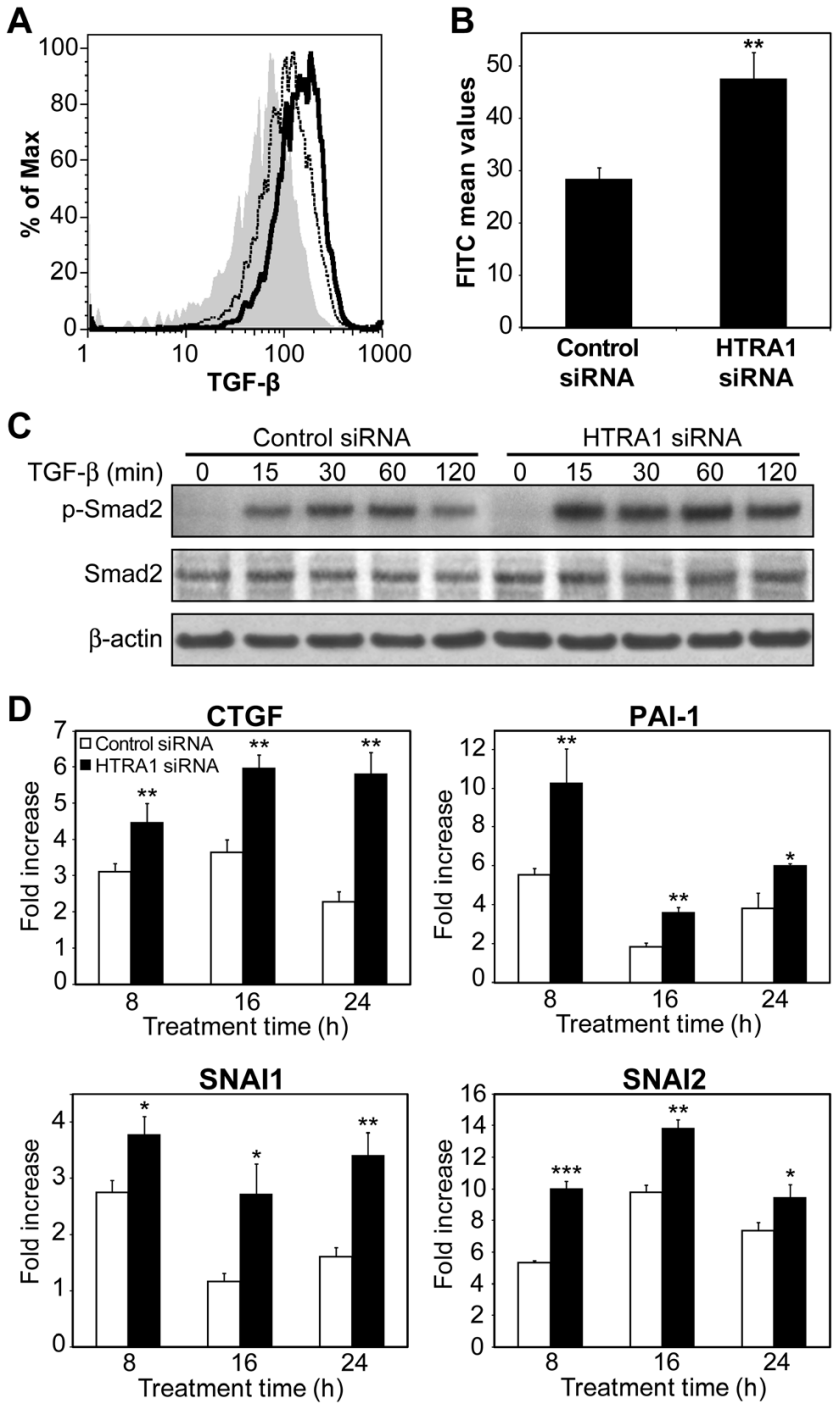
Regulation of TGF-β signaling by HTRA1. (*A*)(*B*) Binding of TGF-β to the cell surface as measured by flow cytometry. Cells were transfected with either nonspecific control siRNA or HTRA1 siRNA and then incubated with either biotinylated TGF-β or a biotinylated negative control protein to measure the background. Solid gray = background, dashed line = control siRNA, black line = HTRA1 siRNA. (*B*) Bar chart indicates the mean FITC values after subtraction of the background level. **P<0.01, Student’s *t*-test, n = 4. (*C*) Immunoblot of total and phosphorylated Smad2 (465/467) in A549 cells transfected with either control or HTRA1 siRNA, and then treated with 1 ng/ml TGF-β for the indicated times. β-actin was used as a loading control. (*D*) The effect of HTRA1 knockdown on the expression of TGF-β-regulated genes. A549 cells were transfected with either nonspecific control siRNA or HTRA1 siRNA and then treated with 1 ng/ml TGF-β for the indicated times. *P<0.05, **P<0.01, ***P<0.001, Student’s *t*-test, n = 3.

We next wanted to demonstrate that the presence of HTRA1 reduced the amount of TGF-β binding to the surface of the cell, so we used flow cytometry to quantify the amount of bound TGF-β in cells transfected with either nonspecific control siRNA or HTRA1 siRNA. Previous work has found that A549 cells are more responsive to TGF-β than HeLa cells [21], therefore we moved forward using these cells. The cells were transfected with HTRA1 siRNA, which reduced HTRA1 protein levels (Figure S2B). Knockdown of HTRA1 led to an approximately 60% increase in binding of TGF-β to the cell surface (Figure 3A and B), providing further evidence that HTRA1 regulates TGF-β signaling at the cell surface.

Binding of TGF-β to its receptor leads to activation of TβRI, which then propagates the signal via the intracellular phosphorylation of receptor-regulated Smad proteins (R-Smads). Following TGF-β stimulation, we found that Smad2 was phosphorylated within 15 min. HTRA1 knockdown resulted in a 1.9-fold increase in phospho-Smad2 levels after 2 h of stimulation (Figure 3C), indicating an increase in TGF-β signaling. Subsequent to phosphorylation, the R-Smad complex translocates into the nucleus and binds to Smad-responsive elements within a promoter, thus regulating gene expression. Therefore, we next examined whether the increase of phosphorylation due to the reduction of HTRA1 also modulated the expression of TGF-β-regulated genes. Following transfection with either control or HTRA1 siRNA, we treated cells with TGF-β and measured the induction of several genes known to be direct transcriptional targets of TGF-β signaling: *CTGF* [26], *PAI-1* [25], *SNAI1* and *SNAI2* [27]. The loss of HTRA1 significantly increased expression of these genes after TGF-β stimulation for 8, 16 and 24 h (Figure 3D). This confirms that receptor cleavage by HTRA1 affects the downstream signaling of TGF-β, and ultimately results in the inhibition of TGF-β-regulated gene expression.

### Knockout of the *HTRA1* Gene Enhances TGF-β Signaling in Mouse Embryonic Fibroblasts and Causes an Increase in Bone Formation in Mice

**Figure 4:**
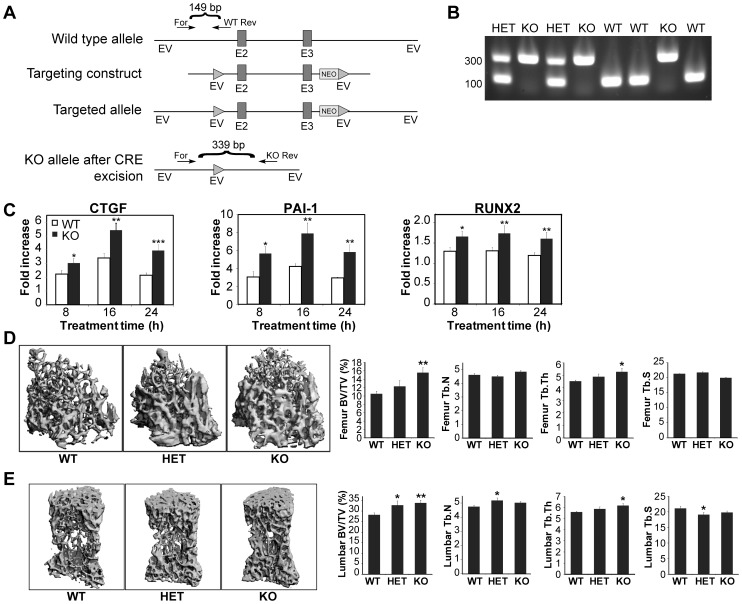
The *in vivo* effects of loss of HTRA1. (*A*) Schematic of the *HTRA1* knockout strategy. Primers were designed with one forward primer (For) that amplifies an amplicon for the wild type and heterozygous alleles with a corresponding reverse primer (Rev), and a second reverse primer designed to only amplify the knockout. The knockout reverse primer (KO Rev) only amplifies following CRE-LOX recombination. Solid triangles = loxP sites; EV = EcoRI. E2 = Exon2; E3 = Exon3. (*B*) Genotype results of mouse embryonic fibroblasts showing confirmation of the generation of *HTRA1*+/− and *HTRA1*−/− mice. (*C*) Expression of TGF-β-regulated genes in embryonic fibroblasts from wild type (WT) and *HTRA1* knockout (KO) mice. Cells were treated with 1 ng/ml TGF-β for the indicated times. *P<0.05, **P<0.01, ***P<0.001, Student’s *t*-test, n = 9 for each cell type. (*D*) Microcomputed tomography and parameters of trabecular bone in the distal femurs in wild type (WT), *HTRA1* heterozygous (HET) and *HTRA1* knockout (KO) mice (all males, 3 months old). (*E*) Microcomputed tomography and parameters of trabecular bone in the fourth vertebrae in wild type (WT), *HTRA1* heterozygous (HET) and *HTRA1* knockout (KO) mice. For both (*D*) and (*E*), *P<0.05, **P<0.01, ***P<0.001, ANOVA and Fisher’s protected least significant difference test, n = 13.

To explore the role of HTRA1 *in vivo*, we generated *HTRA1* null mice. Exons 2 and 3 of the *HTRA1* gene were flanked by LoxP sites, which were deleted following Cre-mediated excision (Figure 4A). This resulted in an open reading frame shift, generating a null mutation. To confirm the deletion, primers were designed such that there was one forward primer and two reverse primers, with the wild type reverse primer located upstream of exon 2 and the knockout reverse primer located after the 3′ LoxP site (Figure 4A). PCR of the wild type allele thus generated an ∼150 bp product, whereas the knockout reverse primer produced an ∼340 bp product that could only be amplified following Cre-Lox recombination (Figure 4B). The heterozygous mice were identified by the presence of both of these bands (Figure 4B).

We then isolated mouse embryonic fibroblasts (MEFs) from both the wild type and *HTRA1−/−* mice, and assessed the changes in expression of TGF-β-regulated genes resulting from the *HTRA1* deletion. Following stimulation with TGF-β for 8, 16, and 24 h, we found that deletion of *HTRA1* significantly enhanced the induction of three genes known to be involved in bone formation: *RUNX2*, *CTGF*, and *PAI-1* (Figure 4C). RUNX2 is a transcription factor that is essential for osteoblast differentiation and bone formation [28]. A conditional gene knockout study has demonstrated that CTGF is necessary for normal skeletal development [29]. PAI-1, a specific inhibitor of two plasminogen activators, tissue-type plasminogen activator (tPA) and urokinase-type plasminogen activator (uPA), may also contribute to the regulation of bone homeostasis, since mice lacking tPA and uPA show increased bone formation and bone mass [30]. Given the effect of HTRA1 deletion on the transcription of osteogenic genes in MEF cells, we used microcomputed tomography (µCT) to examine the trabecular architecture of the vertebrae (Figure 4D) and femurs (Figure 4E) of wild type, heterozygous and *HTRA1* null mice. The results presented show the µCT scans with bar graphs below to indicate the morphometric parameters (bone volume fraction, and trabecular thickness, number, and separation). A significant increase in bone volume in both the distal femurs and the vertebrae was detected in the *HTRA1* knockout mice (by 48.3% and 19.9%, respectively). Trabecular thickness in both the distal femurs and the vertebrae was also found to be significantly increased in the HTRA1 knockout mice (Figure 4D and E). Importantly, there was no difference in body weight between the HTRA1−/− mice and the wild type mice at 3 months of age, and gross examination of the HTRA1−/− mice did not reveal any non-skeletal abnormalities. This suggests that HTRA1 negatively regulates bone mass *in vivo*, possibly by inhibiting TGF-β family signaling.

## Discussion

An inhibitory effect of HTRA1 on the TGF-β family pathway has been previously reported [1], [12], [13], but the precise mechanism of inhibition was not fully understood. Our study demonstrates that HTRA1 antagonizes TGF-β signaling by cleaving its receptors. HTRA1 has been shown to bind to several TGF-β family members, but despite the fact that inhibition of TGF-β family signaling by HTRA1 is dependent on its proteolytic activity, HTRA1 did not appear to cleave these factors [1]. An intracellular mechanism was recently proposed by Shiga et al., who reported that HTRA1 decreased TGF-β signaling by cleaving proTGF-β within the intracellular space, thus reducing the amount of mature TGF-β [12]. They showed that HTRA1 was able to cleave LAP, the N-terminal of proTGF- β, within the endoplasmic reticulum (ER), but that it was unable to cleave mature TGF-β. In contrast, an earlier study showed that HTRA1 was able to inhibit signaling triggered by recombinant mature TGF-β in mouse C2C12 myoblast cells [1]. In this case recombinant TGF- β was added to the culture medium, therefore inhibition could not have occurred via an intracellular event. Because the active form of HTRA1 exists as both a secreted protein [31] as well as an intracytoplasmic protein [32], [33], it seems probable that HTRA1 can regulate TGF-β at different points in the pathway. Our data show that an extracellular regulatory mechanism does in fact exist, whereby HTRA1 mediates degradation of the TGF-β receptors. Our results indicate that HTRA1 cleaves the TGF-β receptors TβRII and TβRIII, but does not affect TβRI, and that this results in a decrease of downstream TGF-β signaling.

TGF-β is known to regulate the bone-remodeling process, although its role is complicated because it impacts both bone resorption and formation [34]. The modulation of TGF-β signaling at the receptor level can have direct consequences in the homeostasis of bone development, as shown by several *in viv*o studies. Conditional knockout of *Tgfbr2* in Col2a-expressing cells resulted in defects in skeletal development of the skull and the vertebrae during the embryonic stage [35], and deletion of *Tgfbr2* by Prx-Cre caused defects in the long bones, joints and skull [36], [37].

HTRA1 has been suggested to play a part in bone development as well, and it has been shown that there is a correlation between the expression patterns of HTRA1 and TGF-β [1], [2]. Indeed, HTRA1 has been shown to have a role in osteogenesis and mineralization, although the data are conflicting as to whether it promotes or inhibits this process. Consistent with our results, an *in vitro* study reported that HTRA1 has a negative impact on mineralization [3]. HTRA1 expression increased in differentiating 2T3 osteoblasts, but was subsequently downregulated following mineralization. SiRNA silencing of HTRA1 expression in these cells resulted in increased mineralization, whereas HTRA1 overexpression reduced it. In contrast, Tiaden et al. found that HTRA1 increased osteogenesis of mesenchymal stem cells (MSCs) and promoted mineralization by differentiating bone cells [38]. Recombinant HTRA1 added to the initiation phase of mineralization promoted formation of the mineralized matrix but had no effect at earlier time points, suggesting that the differentiation status of the cells was important to this regulation. It is possible that these inconsistent *in vitro* results stem from differences in cell type, differentiation stage or culture conditions, similar to what has been shown to occur with TGF-β [34]. Despite the discrepancies in the *in vitro* experiments, our *in vivo* data clearly demonstrate that the end result of a loss of HTRA1 is an increase in bone formation. We have found that deletion of *HTRA1* in mice causes a significant increase in trabecular bone volume, as determined by µCT. By comparing wild type and HTRA1 deficient MEFs, we found that several genes that are repressed by HTRA1 are involved in the regulation of bone formation. These findings reveal a critical role of HTRA1 in regulating bone development.

We hypothesize that HTRA1 may inhibit bone formation by antagonizing signaling mediated by TGF-β family proteins, and by repressing the expression of downstream genes such as *RUNX2*, *CTGF*, and *PAI-1* which are known to be essential during bone development. From our data we cannot conclude that the effect of HTRA1 on bone formation is solely due to inhibition of TGF-β, as antagonism of other TGF-β family members by HTRA1 could contribute to this effect as well. It has been reported that TβRIII directly and specifically binds to some BMPs to enhance their signaling, including BMP-2, BMP-4 and BMP-7, with similar kinetics as previously determined for TGF-β [39]. All three of these BMPs are well known anabolic factors that can potently induce bone formation. Indeed, overexpression of HTRA1 in 2T3 osteoblasts prevents BMP-2-induced mineralization *in vitro* [3], suggesting that HTRA1 may inhibit BMP-2 signaling and matrix calcification by cleaving TβRIII. In additional, HTRA1 may be acting via dual-mechanisms, anti-anabolically by antagonizing signaling pathways mediated by TGF-β family proteins, as well as catabolically by cleaving matrix proteins [13], [16]. It is therefore possible that increased bone mass found in *HTRA1*−/− mice may be in part due to decreased degradation of some bone matrix proteins. Clearly more work is needed to further characterize the mechanisms by which HTRA1 regulates osteogenesis.

## Conclusion

Although HTRA1 is known to bind to TGF-β family members, this is the first study to show that HTRA1 is able to cleave TGF-β receptors. Our results identify a novel mechanism by which HTRA1 inhibits TGF-β signaling, demonstrating that HTRA1 degrades TβRII and TβRIII, but not TβRI, which subsequently leads to decreased downstream TGF-β signaling. Moreover, several genes that are repressed by HTRA1 are involved in the regulation of bone formation, and importantly, deletion of *HTRA1* significantly increased trabecular bone volume. The current study identified a novel mechanism by which HTRA1 inhibits TGF-β signaling, and suggests that HtrA1 may play an important role in bone development, possibly by inhibiting signaling of TGF-β family proteins.

## Supporting Information

Figure S1 **HeLa cells are responsive to TGF-β treatment.** Description: Treatment with TGF-β for the indicated times induced phosphorylation of Smad2, as measured by western blotting.
(TIF)

Figure S2 **HTRA1 knockdown in HeLa and A549 cells.** Description: A) HeLa cells or B) A549 cells were transfected with either a nonspecific control siRNA or an siRNA targeting HTRA1. Knockdown of HTRA1 was analyzed by western blotting. β-actin was used as a loading control. Blots are representative of three independent experiments.
(TIF)

## References

[1] Oka C, Tsujimoto R, Kajikawa M, Koshiba-Takeuchi K, Ina J, et al (2004) HtrA1 serine protease inhibits signaling mediated by Tgfbeta family proteins. Development 131: 1041–1053. 14973287 10.1242/dev.00999

[2] Tsuchiya A, Yano M, Tocharus J, Kojima H, Fukumoto M, et al (2005) Expression of mouse HtrA1 serine protease in normal bone and cartilage and its upregulation in joint cartilage damaged by experimental arthritis. Bone 37: 323–336. 15993670 10.1016/j.bone.2005.03.015

[3] Hadfield KD, Rock CF, Inkson CA, Dallas SL, Sudre L, et al (2008) HtrA1 inhibits mineral deposition by osteoblasts: requirement for the protease and PDZ domains. J Biol Chem 283: 5928–5938. 18156628 10.1074/jbc.M709299200

[4] Dewan A, Liu M, Hartman S, Zhang SS, Liu DT, et al (2006) HTRA1 promoter polymorphism in wet age-related macular degeneration. Science 314: 989–992. 17053108 10.1126/science.1133807

[5] Yang Z, Camp NJ, Sun H, Tong Z, Gibbs D, et al (2006) A variant of the HTRA1 gene increases susceptibility to age-related macular degeneration. Science 314: 992–993. 17053109 10.1126/science.1133811

[6] Vierkotten S, Muether PS, Fauser S (2011) Overexpression of HTRA1 leads to ultrastructural changes in the elastic layer of Bruch’s membrane via cleavage of extracellular matrix components. PLoS One 6: e22959. 21829675 10.1371/journal.pone.0022959 PMC3149070

[7] Hara K, Shiga A, Fukutake T, Nozaki H, Miyashita A, et al (2009) Association of HTRA1 mutations and familial ischemic cerebral small-vessel disease. N Engl J Med 360: 1729–1739. 19387015 10.1056/NEJMoa0801560

[8] Bonewald LF, Mundy GR (1990) Role of transforming growth factor-beta in bone remodeling. Clin Orthop Relat Res: 261–276. 2403492

[9] Tang Y, Wu X, Lei W, Pang L, Wan C, et al (2009) TGF-beta1-induced migration of bone mesenchymal stem cells couples bone resorption with formation. Nat Med 15: 757–765. 19584867 10.1038/nm.1979 PMC2727637

[10] Lopez-Casillas F, Wrana JL, Massague J (1993) Betaglycan presents ligand to the TGF beta signaling receptor. Cell 73: 1435–1444. 8391934 10.1016/0092-8674(93)90368-z

[11] Sankar S, Mahooti-Brooks N (1995) Centrella M, McCarthy TL, Madri JA (1995) Expression of transforming growth factor type III receptor in vascular endothelial cells increases their responsiveness to transforming growth factor beta 2. J Biol Chem 270: 13567–13572. 7768960 10.1074/jbc.270.22.13567

[12] Shiga A, Nozaki H, Yokoseki A, Nihonmatsu M, Kawata H, et al (2011) Cerebral small-vessel disease protein HTRA1 controls the amount of TGF-beta1 via cleavage of proTGF-beta1. Hum Mol Genet 20: 1800–1810. 21320870 10.1093/hmg/ddr063

[13] Tocharus J, Tsuchiya A, Kajikawa M, Ueta Y, Oka C, et al (2004) Developmentally regulated expression of mouse HtrA3 and its role as an inhibitor of TGF-beta signaling. Dev Growth Differ 46: 257–274. 15206957 10.1111/j.1440-169X.2004.00743.x

[14] Lengner CJ, Lepper C, van Wijnen AJ, Stein JL, Stein GS, et al (2004) Primary mouse embryonic fibroblasts: a model of mesenchymal cartilage formation. J Cell Physiol 200: 327–333. 15254959 10.1002/jcp.20118

[15] Murwantoko, Yano M, Ueta Y, Murasaki A, Kanda H, et al (2004) Binding of proteins to the PDZ domain regulates proteolytic activity of HtrA1 serine protease. Biochem J 381: 895–904. 15101818 10.1042/BJ20040435 PMC1133901

[16] Chamberland A, Wang E, Jones AR, Collins-Racie LA, LaVallie ER, et al (2009) Identification of a novel HtrA1-susceptible cleavage site in human aggrecan: evidence for the involvement of HtrA1 in aggrecan proteolysis in vivo. J Biol Chem 284: 27352–27359. 19657146 10.1074/jbc.M109.037051 PMC2785663

[17] Rüegsegger P, Koller B, Muller R (1996) A microtomographic system for the nondestructive evaluation of bone architecture. Calcif Tissue Int 58: 24–29. 8825235 10.1007/BF02509542

[18] Hildebrand T, Rüegsegger P (1997) A new method for the model independent assessment of thickness in three dimensional images. Journal of Microscopy 185: 67–75.

[19] Odgaard A (1997) Three-dimensional methods for quantification of cancellous bone architecture. Bone 20: 315–328. 9108351 10.1016/s8756-3282(97)00007-0

[20] Hildebrand T, Laib A, Muller R, Dequeker J, Ruegsegger P (1999) Direct three-dimensional morphometric analysis of human cancellous bone: microstructural data from spine, femur, iliac crest, and calcaneus. J Bone Miner Res 14: 1167–1174. 10404017 10.1359/jbmr.1999.14.7.1167

[21] Kim YW, Park J, Lee HJ, Lee SY, Kim SJ (2012) TGF-beta sensitivity is determined by N-linked glycosylation of the type II TGF-beta receptor. Biochem J 445: 403–411. 22571197 10.1042/BJ20111923 PMC3462611

[22] Van Themsche C, Chaudhry P, Leblanc V, Parent S, Asselin E (2010) XIAP gene expression and function is regulated by autocrine and paracrine TGF-beta signaling. Mol Cancer 9: 216. 20712893 10.1186/1476-4598-9-216 PMC2933620

[23] Murakami M, Kawachi H, Ogawa K, Nishino Y, Funaba M (2009) Receptor expression modulates the specificity of transforming growth factor-beta signaling pathways. Genes Cells 14: 469–482. 19335617 10.1111/j.1365-2443.2009.01283.x

[24] Hu SI, Carozza M, Klein M, Nantermet P, Luk D, et al (1998) Human HtrA, an evolutionarily conserved serine protease identified as a differentially expressed gene product in osteoarthritic cartilage. J Biol Chem 273: 34406–34412. 9852107 10.1074/jbc.273.51.34406

[25] Westerhausen DR Jr, Hopkins WE, Billadello JJ (1991) Multiple transforming growth factor-beta-inducible elements regulate expression of the plasminogen activator inhibitor type-1 gene in Hep G2 cells. J Biol Chem 266: 1092–1100. 1985937

[26] Grotendorst GR, Okochi H, Hayashi N (1996) A novel transforming growth factor beta response element controls the expression of the connective tissue growth factor gene. Cell Growth Differ 7: 469–480. 9052988

[27] Peinado H, Quintanilla M, Cano A (2003) Transforming growth factor beta-1 induces snail transcription factor in epithelial cell lines: mechanisms for epithelial mesenchymal transitions. J Biol Chem 278: 21113–21123. 12665527 10.1074/jbc.M211304200

[28] Ducy P, Zhang R, Geoffroy V, Ridall AL, Karsenty G (1997) Osf2/Cbfa1: a transcriptional activator of osteoblast differentiation. Cell 89: 747–754. 9182762 10.1016/s0092-8674(00)80257-3

[29] Canalis E, Zanotti S, Beamer WG, Economides AN, Smerdel-Ramoya A (2010) Connective tissue growth factor is required for skeletal development and postnatal skeletal homeostasis in male mice. Endocrinology 151: 3490–3501. 20534727 10.1210/en.2010-0145 PMC2940511

[30] Daci E, Everts V, Torrekens S, Van Herck E, Tigchelaar-Gutterr W, et al (2003) Increased bone formation in mice lacking plasminogen activators. J Bone Miner Res 18: 1167–1176. 12854826 10.1359/jbmr.2003.18.7.1167

[31] Zumbrunn J, Trueb B (1996) Primary structure of a putative serine protease specific for IGF-binding proteins. FEBS Lett 398: 187–192. 8977104 10.1016/s0014-5793(96)01229-x

[32] Clawson GA, Bui V, Xin P, Wang N, Pan W (2008) Intracellular localization of the tumor suppressor HtrA1/Prss11 and its association with HPV16 E6 and E7 proteins. J Cell Biochem 105: 81–88. 18452160 10.1002/jcb.21804

[33] Chien J, Ota T, Aletti G, Shridhar R, Boccellino M, et al (2009) Serine protease HtrA1 associates with microtubules and inhibits cell migration. Mol Cell Biol 29: 4177–4187. 19470753 10.1128/MCB.00035-09 PMC2715801

[34] Janssens K, ten Dijke P, Janssens S, Van Hul W (2005) Transforming growth factor-beta1 to the bone. Endocr Rev 26: 743–774. 15901668 10.1210/er.2004-0001

[35] Baffi MO, Slattery E, Sohn P, Moses HL, Chytil A, et al (2004) Conditional deletion of the TGF-beta type II receptor in Col2a expressing cells results in defects in the axial skeleton without alterations in chondrocyte differentiation or embryonic development of long bones. Dev Biol 276: 124–142. 15531369 10.1016/j.ydbio.2004.08.027

[36] Seo HS, Serra R (2007) Deletion of Tgfbr2 in Prx1-cre expressing mesenchyme results in defects in development of the long bones and joints. Dev Biol 310: 304–316. 17822689 10.1016/j.ydbio.2007.07.040 PMC2042108

[37] Seo HS, Serra R (2009) Tgfbr2 is required for development of the skull vault. Dev Biol 334: 481–490. 19699732 10.1016/j.ydbio.2009.08.015 PMC2753698

[38] Tiaden AN, Breiden M, Mirsaidi A, Weber FA, Bahrenberg G, et al (2012) Human serine protease HTRA1 positively regulates osteogenesis of human bone marrow-derived mesenchymal stem cells and mineralization of differentiating bone-forming cells through the modulation of extracellular matrix protein. Stem Cells 30: 2271–2282. 22865667 10.1002/stem.1190

[39] Kirkbride KC, Townsend TA, Bruinsma MW, Barnett JV, Blobe GC (2008) Bone morphogenetic proteins signal through the transforming growth factor-beta type III receptor. J Biol Chem 283: 7628–7637. 18184661 10.1074/jbc.M704883200

