# Supplementary figures and images for: Serine Protease HTRA1 Antagonizes Transforming Growth Factor-β Signaling by Cleaving Its Receptors and Loss of HTRA1 In Vivo Enhances Bone Formation
Source: PLoS One. 2013 Sep 11;8(9):e74094. doi: 10.1371/journal.pone.0074094 (PMC3770692; doi:10.1371/journal.pone.0074094)

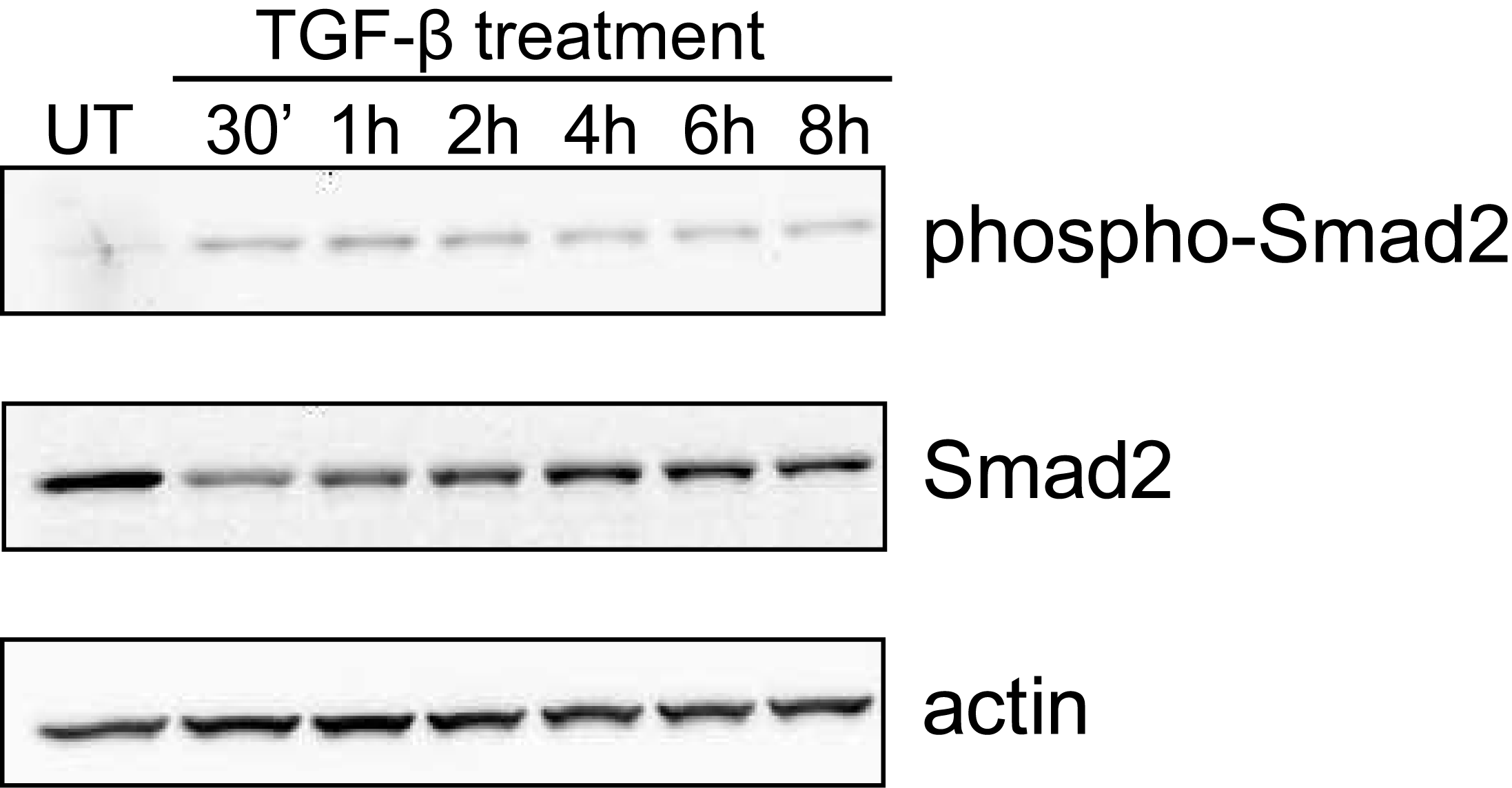

Supplement: Figure S1 — HeLa cells are responsive to TGF-β treatment. Description: Treatment with TGF-β for the indicated times induced phosphorylation of Smad2, as measured by western blotting. (TIF) [file pone.0074094.s001.tif]

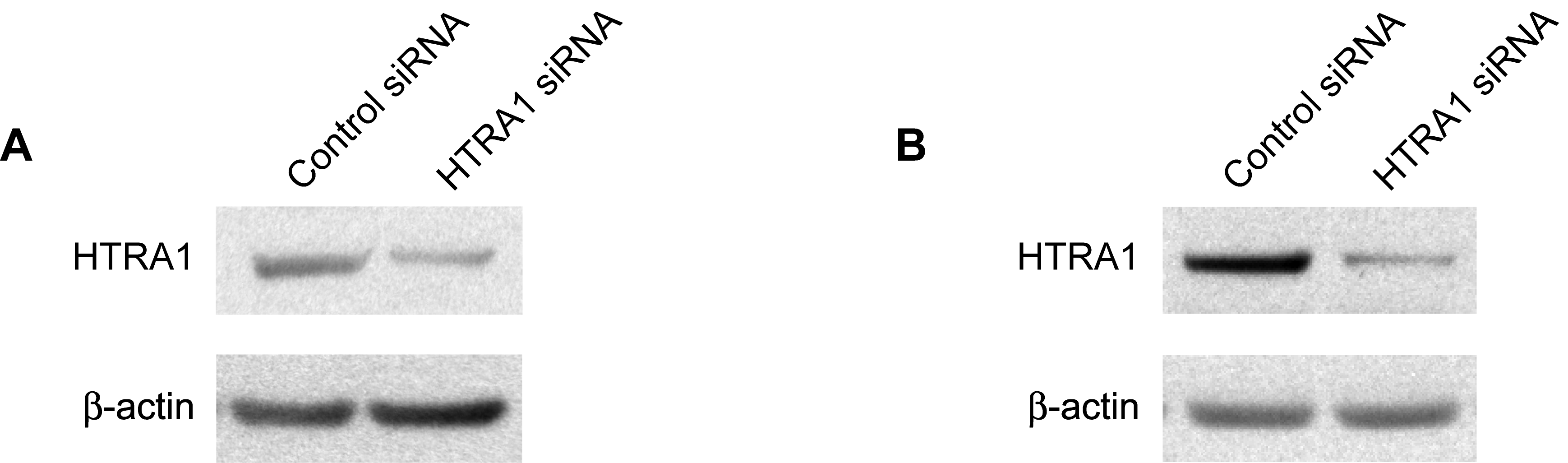

Supplement: Figure S2 — HTRA1 knockdown in HeLa and A549 cells. Description: A) HeLa cells or B) A549 cells were transfected with either a nonspecific control siRNA or an siRNA targeting HTRA1. Knockdown of HTRA1 was analyzed by western blotting. β-actin was used as a loading control. Blots are representative of three independent experiments. (TIF) [file pone.0074094.s002.tif]
